# Influenza Screening via Deep Learning Using a Combination of Epidemiological and Patient-Generated Health Data: Development and Validation Study

**DOI:** 10.2196/21369

**Published:** 2020-10-29

**Authors:** Hyunwoo Choo, Myeongchan Kim, Jiyun Choi, Jaewon Shin, Soo-Yong Shin

**Affiliations:** 1 Department of Digital Health Samsung Advanced Institute for Health Sciences and Technology Sungkyunkwan University Seoul Republic of Korea; 2 Mobile Doctor Seoul Republic of Korea; 3 Big Data Research Center Samsung Medical Center Seoul Republic of Korea

**Keywords:** influenza, screening tool, patient-generated health data, mobile health, mHealth, deep learning

## Abstract

**Background:**

Screening for influenza in primary care is challenging due to the low sensitivity of rapid antigen tests and the lack of proper screening tests.

**Objective:**

The aim of this study was to develop a machine learning–based screening tool using patient-generated health data (PGHD) obtained from a mobile health (mHealth) app.

**Methods:**

We trained a deep learning model based on a gated recurrent unit to screen influenza using PGHD, including each patient’s fever pattern and drug administration records. We used meteorological data and app-based surveillance of the weekly number of patients with influenza. We defined a single episode as the set of consecutive days, including the day the user was diagnosed with influenza or another disease. Any record a user entered 24 hours after his or her last record was considered to be the start of a new episode. Each episode contained data on the user’s age, gender, weight, and at least one body temperature record. The total number of episodes was 6657. Of these, there were 3326 episodes within which influenza was diagnosed. We divided these episodes into 80% training sets (2664/3330) and 20% test sets (666/3330). A 5-fold cross-validation was used on the training set.

**Results:**

We achieved reliable performance with an accuracy of 82%, a sensitivity of 84%, and a specificity of 80% in the test set. After the effect of each input variable was evaluated, app-based surveillance was observed to be the most influential variable. The correlation between the duration of input data and performance was not statistically significant (*P*=.09).

**Conclusions:**

These findings suggest that PGHD from an mHealth app could be a complementary tool for influenza screening. In addition, PGHD, along with traditional clinical data, could be used to improve health conditions.

## Introduction

With the increasing popularity of mobile health (mHealth), a considerable amount of health-related data are now generated and accumulated outside of hospitals [1-3]. These health-related data cover a wide range of quantitative variables, such as physical activity, blood glucose levels, blood pressure, heart rate/rhythm, and oxygen saturation along with a range of qualitative data, such as mood-related symptoms, food intake, medication use, and sleep patterns. Even data from social media posts or search engine queries may be included [4]. These kinds of health-related data are categorized as patient-generated health data (PGHD) and defined by the Office of the National Coordinator for Health Information Technology as “health-related data—including health history, symptoms, biometric data, treatment history, lifestyle choices, and other information—created, recorded, gathered, or inferred by or from patients or their designees (i.e., care partners or those who assist them) to help address a health concern” [5].

Many studies have shown that PGHD have various potential benefits for health care. For example, PGHD may help patients with chronic diseases like diabetes or hypertension take better care of themselves by delivering continuous monitoring and support with more personalized treatment planning [6-9]. PGHD are also beneficial for remote monitoring of patients’ postsurgical pain or chronic pain and have been found to more accurately assess the psychoemotional status of patients [10-12]. Another example of PGHD use is forecasting contagious diseases. Some research has shown that influenza [13-15] and Middle East respiratory syndrome (MERS) [16] outbreaks could be predicted using search engine query data, including Google Flu Trends and social media posts. In addition to these indirect methods, a website or smartphone app through which patients directly report their symptoms can also be used to detect epidemics [17,18].

Although influenza outbreaks can be predicted using PGHD, the diagnosis or screening of individual patients has been conducted using traditional medical devices, such as the rapid influenza antigen test or reverse transcription–polymerase chain reaction (RT-PCR). The rapid influenza diagnostic test (RIDT) has mainly been used as a diagnostic test because of its reduced processing time and easy accessibility [19]. However, due to the low sensitivity of the RIDT, it is insufficient to serve as a screening test for influenza [20-22]. Due to this concern, influenza treatment with antiviral medication has been prescribed for suspected influenza cases, based on clinical judgment, even when the RIDT showed a negative result. Influenza-like illness (ILI) case definition is one of the symptom-based screening methods of suspected cases, but it has been reported to have limited sensitivity despite its loss of substantial specificity [23].

Fever is regarded as the most distinctive symptom of influenza. Due to the lack of other distinguishable symptoms, it can be challenging to differentiate influenza from other diseases [24,25]. Recently, deep learning approaches have been reported to exceed classical statistical methods for predicting the outcomes of an individual patient using time series data, such as inpatient data [23,26]. In this study, we propose a deep learning method for influenza screening by combining epidemiological information and PGHD from an mHealth app. These results were then compared with the patients’ diagnostic findings.

## Methods

### Data Collection

**Figure 1:**
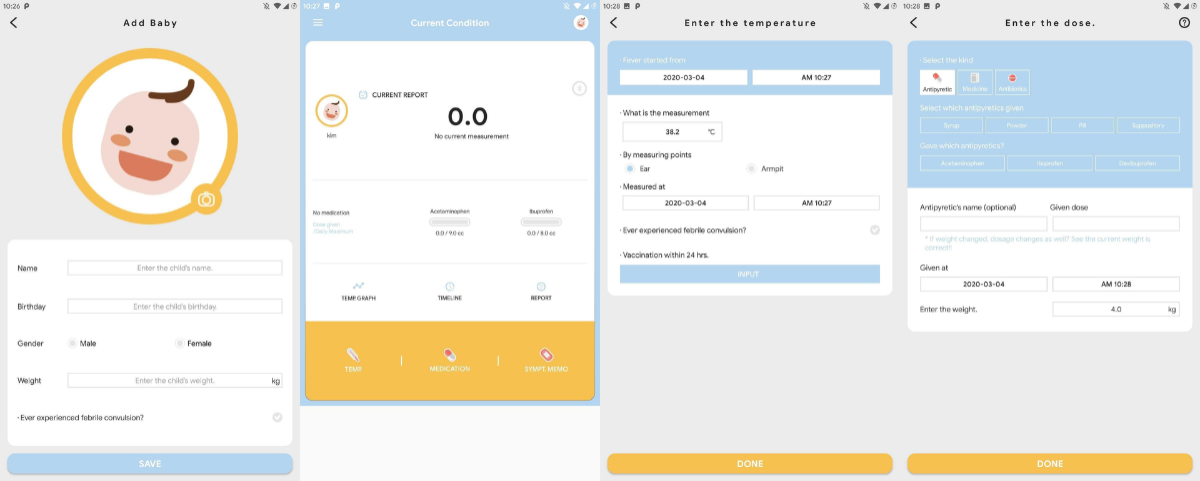
Screenshots of the Fever Coach app.

We retrospectively collected log data from the Fever Coach app, which is available on Android and iOS [27]. Fever Coach is a fever management app that uses the self-reported data of its users (Figure 1).

The data were collected from January 2017 to December 2018. A total of 480,793 users entered 28,010,112 records. During the same period, the number of users diagnosed with influenza at a clinic was 16,432. In 2017 and 2018, 3583 and 12,849 users were diagnosed with influenza, respectively. The log data included body temperature, volume, type and form of antipyretic drugs or antibiotic drugs, sex, age, weight, symptoms, and memos. The users of Fever Coach agreed that their deidentified data could be used for research purposes, and the institutional review board of Samsung Medical Center waived informed consent.

We collected the daily mean temperature, daily maximum temperature, daily minimum temperature, daily mean dew point, daily mean relative humidity, and daily mean pressure data between January 2017 and December 2018 from the Korea Meteorological Administration information portal. The observation point was Seoul 108 [28].

Korea Center for Disease Control (KCDC) produces a weekly influenza-like illness report every Tuesday using data received from public health centers during the previous week. These data were collected for the period of January 2017-December 2018 [29].

### Data Preprocessing

All of the log data, separated by user ID and year, were then split into episodes. The episodes were defined as the set of consecutive days containing the day the user was diagnosed with influenza or another disease. For example, if a user was diagnosed with influenza on February 23, 2018, and recorded his or her body temperature between February 21, 2018, and February 24, 2018, these days were considered to be 1 episode. If the user logged another record 24 hours after his or her previous record, it was considered to be a new episode. Table 1 shows examples of episode separation.

Each episode must contain information about the user’s age, gender, and weight. Users were divided into 4 age groups—0-2 years, 2-5 years, 6-12 years, and ≥13 years—to avoid possible overfitting according to age, as age is one of the key factors of influenza propagation. Any episode without age, gender, and weight was excluded. Moreover, any episode not containing at least 1 fever data point was excluded.

We then calculated the app-based weekly influenza surveillance from the influenza-diagnosed episodes each year. The app-based weekly influenza surveillance was defined by the weekly number of reported influenza cases divided by the total number of annually reported influenza cases in the same year. For example, if there were 3000 reported influenza cases in 2018 and 300 weekly reported influenza cases in week 49 of 2018, the app-based surveillance for week 49 of 2018 was 0.1. We calculated this value every week for each year and then added this value to the corresponding episode. If each episode had multiple days, we used the first day of each episode as the representative value, considering that the incubation period of influenza is 1 to 4 days [30,31]. Our week-numbering was based on the ISO week-date system [32]. The app-based weekly influenza surveillance data are in Multimedia Appendix 1.

We also added meteorological data from the Korean Meteorological Administration. As before, we used values corresponding to the first day of each episode. We added KCDC laboratory surveillance as well, but this time we used values corresponding to 1 week before the first day of each episode. Due to the reporting delay of the KCDC surveillance, we could not use values corresponding to the same week.

**Figure 2:**
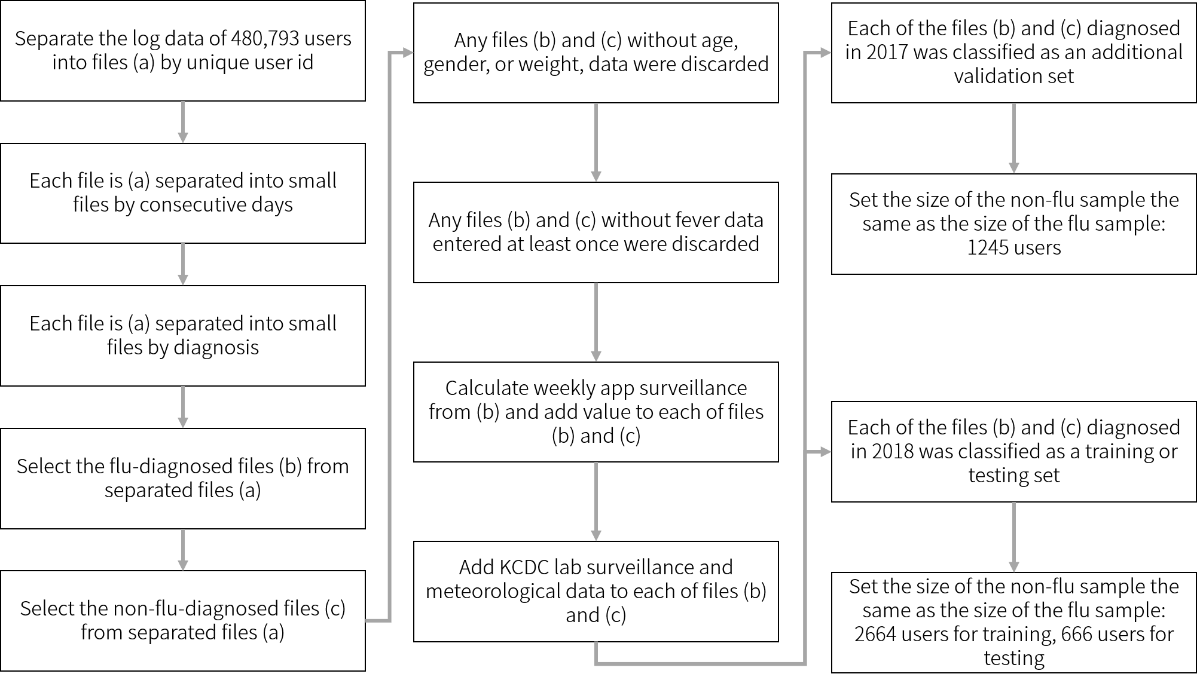
Pipeline for data preprocessing. KCDC: Korea Center for Disease Control.

Finally, as the log data we collected had more noninfluenza episodes than the influenza episodes, we set the number of the noninfluenza episodes to be the same as the influenza episodes each year. Data from 2018 were used for training and hyperparameter tuning, and those data were randomly split into the training set (2664/3330, 80%) and the test set (666/3330, 20%). A 5-fold cross-validation was used on the training set. Considering that the influenza epidemic is slightly different each year, we prepared an additional validation set. Although our training/test sets included the data collected in 2018, the additional validation set included the data collected in 2017 that had a different distribution of weekly reported influenza cases. As with the training/test set, the additional validation set was also adjusted to 50:50 for influenza and noninfluenza episodes. Figure 2 summarizes the overall pipeline for data preprocessing.

**Table 1:** Examples of episode separation.

Episodes and the user-added date and time log		Time elapsed since the previous log	
**Episode 1**			
	2018-09-06 22:25		N/A^a^
	2018-09-06 22:37		0 h 12 min
	2018-09-06 23:53		0 h 16 min
	2018-09-07 1:01		0 h 8 min
	2018-09-07 2:49		1 h 48 min
	2018-09-07 10:00		7 h 11 min
	2018-09-07 15:56		5 h 56 min
	2018-09-07 21:15		5 h 19 min
	2018-09-08 11:20		14 h 5 min
	2018-09-08 12:10		0 h 50 min
	2018-09-08 21:10		9 h 0 min
	2018-09-09 12:14		15 h 4 min
	2018-09-09 21:38		9 h 24 min
	2018-09-10 9:40		12 h 2 min
	2018-09-10 21:30		11 h 50 min
	2018-09-11 9:14		11 h 44 min
	2018-09-11 19:14		10 h 0 min
**Episode** **2**			
	2018-10-03 22:11		> 24 h
	2018-10-03 22:12		0 h 1 min
	2018-10-03 22:26		0 h 14 min
	2018-10-03 23:31		1 h 5 min
	2018-10-04 0:31		1 h 0 min
	2018-10-04 2:38		2 h 7 min
**Episode 3**			
	2018-10-11 8:30		> 24 h
	2018-10-11 10:10		1 h 40 min
	2018-10-11 10:12		0 h 2 min
	2018-10-11 10:14		0 h 2 min
	2018-10-11 11:35		1 h 21 min

^a^N/A: not applicable.

### Deep Learning Model and Training Hyperparameters

We used GRU-D as our baseline model [26]. GRU-D is a modified design of the gated recurrent unit (GRU) neural network structure based on a recurrent neural network. Unlike in the GRU, the mask and timestamp were combined together, and input was manipulated to 3-channel data. Since Fever Coach data were characterized by a variety of missing values, we considered that the mask system of the GRU-D structure would be effective in our experiment. Backpropagation was not performed for the masked data; therefore, it did not update parameters. The input data were manipulated to 3-channel data, which were concatenated with a timestamp and masked as previously described. Thus, the shape of the matrix X_input_ was 3 × D × T, where D is the number of variables for each experiment, and T is the maximum number of time series. We used T=70 in the experiment in that the maximum count of the input data in 1 episode was 70. The maximum number of variable dimensions in our experiment was 16 (4 for age, 6 for meteorological data, and 1 each for sex, weight, influenza surveillance, app-based surveillance, antibiotic administration, and antipyretics administration). We performed 3 experiments using different combinations of variables. First, we used the entire 16 dimensions (7 variables) for inputting the model, and 2 additional experiments were performed to evaluate the effect of the input variables on performance. The second experiment was performed with the same conditions as the first, except a single variable was removed, which brought the number of variables to 6. The third experiment was similar to the first as well, except for the addition of 1 variable out of the 3 (body temperature, antipyretics administration, and antibiotic administration). We used binary cross-entropy as a loss function, and we used accuracy as an evaluation metric to choose the best model. All hidden states were initialized to 0. We used the optimizer, rectified adaptive moment estimation, with a learning rate of 0.0001 [33]. The total number of epochs was 50. The softmax function was used as an activation function. We used a dropout of 0.01 to prevent overfitting. All the input variables were normalized to have a mean of 0 (SD 1). The codes are publicly available at a GitHub repository [34].

## Results

The total number of episodes obtained was 6657. Out of these 6657 episodes, 3326 were diagnosed with influenza. The average and SD of each episode length were 29.24 (SD 21.79). Table 2 summarizes the general characteristics of the processed data.

**Table 2:** General characteristics of the data set.

Variables		Year 2017	Year 2018
**Body temperature**			
	Average number of inputs	15.05	20
	Variance in the number of inputs	16.32	18.29
**Antipyretic administration**			
	Average number of inputs	4.578	6.040
	Variance in the number of inputs	4.685	24.03
**Antibiotic administration, n**			
	At least 1 antibiotic administration	372	4705
	No antibiotic administration	2118	1952
**Age (years), n**			
	0 to 2	886	2529
	2 to 5	1328	3564
	5 to 12	262	479
	Older than 12	14	85
**Sex, n**			
	Male	1246	3348
	Female	1244	3309

**Figure 3:**
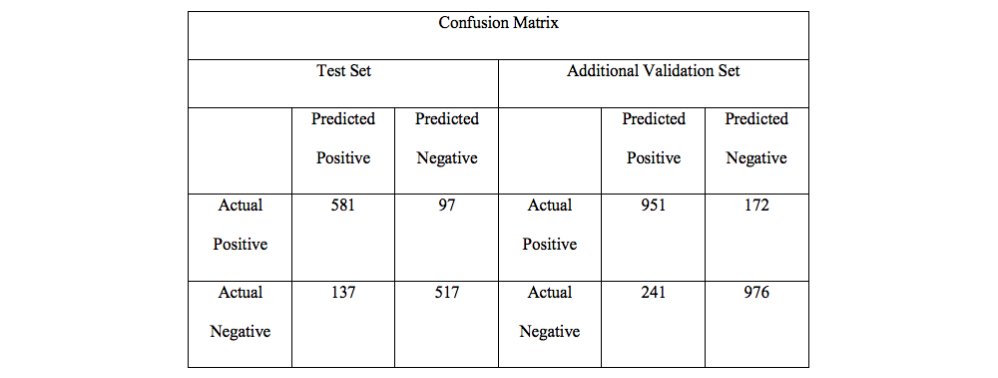
Confusion matrix for the test set and the additional validation set.

**Figure 4:**
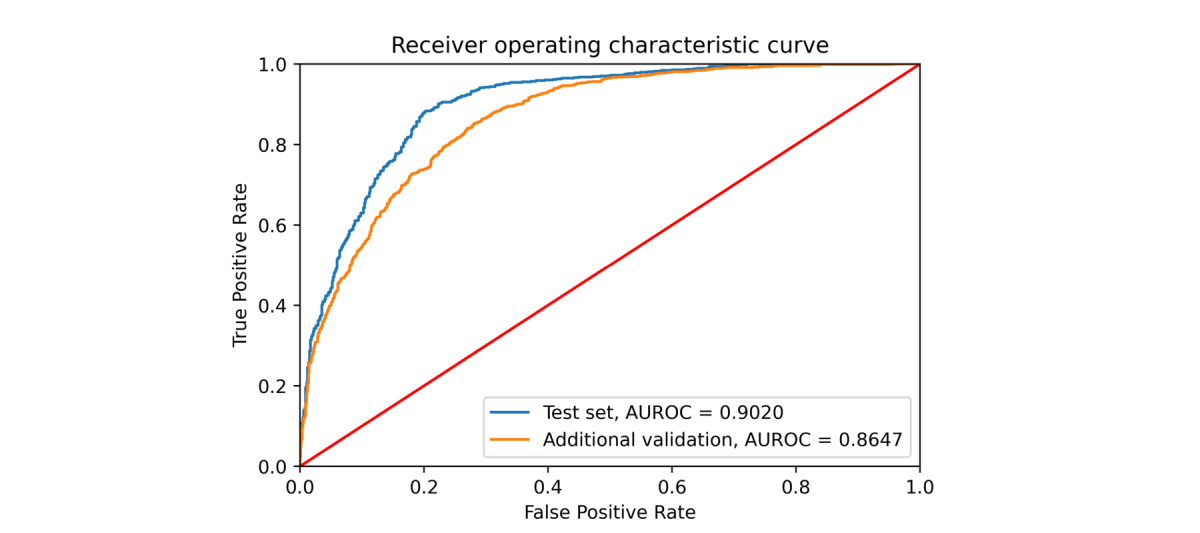
Receiver operating characteristic (ROC) curve illustrating the screening ability of the model. The red line shows a random guess, the blue line is the result of the test set collected in 2018, and the orange line is the result of additional validation using data from 2017. AUROC curve: area under the receiver operating characteristic curve.

Based on the GRU-D, the proposed screening algorithm used PGHD (body temperature records, antipyretic drug administration records, and antibiotic drug administration records), app-based surveillance, and meteorological data as the input variables. The area under the receiver operating characteristic (AUROC) curve of the test data set was 0.902, with an accuracy of 82.43% (95% CI 80.28%-84.44%), a sensitivity of 84.20% (95% CI 81.07%-87.00%), a specificity of 80.92% (95% CI 77.85%-83.73%), a positive predictive value (PPV) of 79.05% (95% CI 76.38%-81.50%), and a negative predictive value (NPV) of 85.69% (95% CI 83.26%-87.83%). The confusion matrix and the receiver operating characteristic (ROC) curve are shown in Figures 3 and 4, respectively.

Considering that the influenza epidemic is slightly different each year, we prepared additional validation set as described in the methods section. For the additional validation set, we achieved an area under the curve (AUC) of 0.8647, an accuracy of 77.99% (95% CI 76.31%-79.61%), a sensitivity of 82.35% (95% CI 79.91%-84.61%), a specificity of 74.79% (95% CI 72.46%-77.02%), a PPV of 70.57% (95% CI 68.59%-72.47%), and an NPV of 85.24% (95% CI 83.47%-86.84%).

We also attempted to evaluate the effect of the input variables on performance in 2 ways. First, we removed them one at a time from all variables. Second, we added them one at a time from baseline variables. To remove them one by one, we first trained the model using all 10 input variables and measured the performance at that time. We then removed 1 input variable and trained the model on the same data set using a total of 9 input variables and measured the performance. We obtained a total of 10 results and summarized them in Table 3. For example, the second row means all variables except fever were used. As a result, the app-based surveillance turned out to be the most influential variable, even though it had little effect on specificity. The second most influential variable was the meteorological observation data. Interestingly, KCDC surveillance data did not seem to have a significant impact. The meteorological factors and app-based surveillance seemed to compensate for the exclusion of the KCDC surveillance data from the input variables.

**Table 3:** The effects of the removal of each variable from the analysis. “–<Variable>” means that the variable was singularly removed from the list of variables for the corresponding experiment.

Variable	Sensitivity	Specificity	AUROC^a^	Accuracy	NPV^b^	F_1_
All	0.8171	0.8425	0.8931	0.8296	0.8163	0.8300
–Sex	0.8510	0.8028	0.8960	0.8273	0.8387	0.8338
–Weight	0.8171	0.8150	0.8832	0.8161	0.8113	0.8189
–Age	0.8333	0.8346	0.8911	0.8339	0.8333	0.8339
–Fever	0.8083	0.8287	0.8882	0.8183	0.8065	0.8191
–Antipyretics	0.8510	0.8058	0.8744^c^	0.8288	0.8392	0.8350
–Anti-viral agent	0.8304	0.8211	0.8892	0.8258	0.8236	0.8292
–App-based surveillance	0.8215	0.7905	0.8775	0.8063^c^	0.8103	0.8120^c^
–KCDC^d^ surveillance	0.8614	0.7813^c^	0.8892	0.8221	0.8446	0.8313
–Meteorological	0.7950^c^	0.8486	0.8900	0.8213	0.7997^c^	0.8191

^a^AUROC: area under the receiver operating characteristic.

^b^NPV: negative predictive value.

^c^The highest decrease in the value for the corresponding column.

^d^KCDC: Korea Center for Disease Control.

Another experiment was conducted to observe the performance changes by defining the base features and adding the variables one at a time (Table 4). The baseline features used were body temperature and the antipyretic and antibiotic drug data. We repeated the analysis by adding each variable to the base features and observing the performance. In each experiment, a total of 4 input variables was used. Consequently, gender data were found to slightly decrease the AUC performance (–0.02), but there was no significant difference between the baseline performance and the performance modified by the addition of gender. Weight and age also displayed no significant differences. For the variables of meteorological data, app surveillance, and KCDC laboratory surveillance, each significantly improved the performance (*P*<.001). There was no significant difference between the performance of "baseline features + app surveillance" and that of "baseline features + meteorological data" (*P*=.48). Similarly, there was no significant difference between the performance of "baseline features + app surveillance" and that of "baseline features + KCDC laboratory survellance" (*P*=.46)

**Table 4:** Effect of each variable on the analysis. The baseline included body temperature, antipyretic drug, and antibiotic drug data. “+<variable>” means that the variable was added to the baseline for the analysis and then removed for the next analysis (noncumulative addition).

Variable	Sensitivity	Specificity	AUROC^a^	Accuracy	NPV^b^	F_1_
Baseline	0.6018	0.7187	0.7221	0.6592	0.6351	0.6425
+sex	0.5678	0.7401	0.7087	0.6524	0.6229	0.6245
+weight	0.5734	0.7523	0.7232	0.6619	0.6332	0.6315
+age	0.5634	0.7477	0.7201	0.6539	0.6229	0.6237
+app surveillance	0.8673^c^	0.7599	0.8808^c^	0.8146^c^	0.8467^c^	0.8264^c^
+KCDC^d^ surveillance	0.7670	0.7936^c^	0.8607	0.7800	0.7666	0.7802
+meteorological	0.8127	0.7470	0.8712	0.7802	0.7961	0.7888

^a^AUROC: area under the receiver operating characteristic.

^b^NPV: negative predictive value.

^c^The highest increase in the value for the corresponding column.

^d^KCDC: Korea Center for Disease Control.

**Figure 5:**
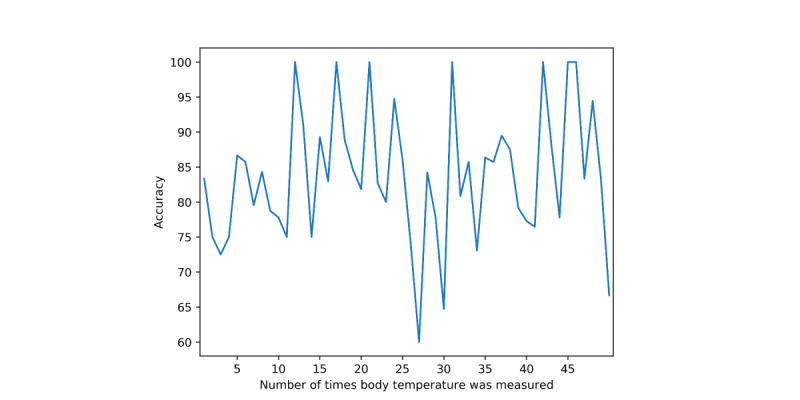
Screening performance versus the number of body temperature records. The y-axis shows the percentage of accuracy, and the x-axis refers to the number of body temperatures entered by the user.

Finally, we looked at the correlation between the duration of the input data and the screening performance. Figure 5 describes the association between the duration of body temperature records and the screening performance. We initially assumed that the prediction would be more accurate if the user entered more data. However, in reality, no correlation was found between the duration of the input data and the screening performance. Spearman rank correlation coefficient was 0.0916. Thus, the association was not considered to be statistically significant.

## Discussion

With this study, we investigated the possibility of screening for influenza using PGHD, such as body temperature and medication records collected from an mHealth app.

At the beginning of this study, we did not know whether body temperature would change when antipyretics were administered, or if body temperature alone was more important. Although fever is a major symptom of influenza, it is impossible to diagnose influenza using only body temperature changes [24,25]. Therefore, we hypothesized that patients with influenza would respond more slowly to antipyretics. To test this hypothesis, we specifically looked at the difference between the performance of the model with and without antipyretic administration. There was a greater change in performance when the antipyretic dose records were removed from the input variable than when only the body temperature was removed. Based on these results, we conclude that the model works as expected. Antibiotic administration records are another variable that we considered important. We expected that the antibiotic administration records and antipyretic administration records would have similar effects, but antibiotic administration records appeared to limit the performance. This might have been due to the ineffectiveness of antibiotics or unnecessary prescription of antibiotics. In our data, 1952 of all 6657 users were prescribed antibiotics, and 674 of those who were prescribed drugs were diagnosed with diseases other than influenza.

Body temperature is known to be one of the most important symptoms of influenza. However, its effect on the model was not as strong as we expected. A temperature higher than 38.3 ºC was recorded at least once during 97.42% (6485/6657) episodes in our data. This shows that the majority of users used the app when their children had a fever, which was the original purpose of the app. Among the episodes, 50.82% (3296/6485) were those of influenza, and 49.18% (3189/6485) were due to other conditions. The mean and variance of body temperature in the patient group diagnosed with influenza were 38.1519 ºC and 0.8611 ºC, respectively; and the mean and variance of body temperature with other conditions were 38.0449º C and 0.8367 ºC. There was a significant difference between the 2 groups (*P*<.001). We speculate that because the app focused on fever, the predictive power of body temperature for influenza was diminished.

One interesting finding was the effect that sex had on specificity. Although some studies have shown that there is a difference in influenza prevalence by gender, our data found that the sex ratio was almost equal, with 1677 males and 1660 females diagnosed with influenza. Moreover, when we excluded sex from the input variables, the accuracy and F_1_ measure did not significantly change. We obtained similar results by repeating the ablation study. Therefore, further research may be needed to clarify this point.

In summary, age, weight, and gender had little effect on the screening performance. App-based surveillance has greatly improved the screening performance and is nearly identical to using KCDC laboratory surveillance or meteorological data, which are frequently used as indicators of influenza outbreaks.

This study has several limitations. First, the training and validation data used were self-reported by the patients. Most users reported their diagnosis using their smartphones; thus, these data were not reported by clinicians. Therefore, we cannot ascertain that the same results would be recorded if hospital-generated data were used. Also, primary care doctors usually use the RIDT instead of RT-PCR to diagnose influenza. As the RIDT has low reliability, our ground truth label may be noisy. For the deep learning model, if the character of the data on deployment is slightly different from that of the training data, it is difficult to achieve the expected performance on validation due to the difficulties in analyzing the effect of the data distribution and input variables on the model [35]. Since the data did not include laboratory results, they are difficult to use in a clinical setting or for general epidemiological analysis; and we expect that the application of limited screening tests through the Fever Coach app will be possible with further research. We are planning to conduct a prospective observational study to address these limitations. Second, various methods were used to measure body temperature. Some of the app users used axillary instead of tympanic temperatures. As there are no primary blood vessels in the axilla, the axillary temperatures are less accurate. This may have influenced the performance of the model.

Screening for influenza can be challenging due to the low sensitivity of rapid antigen tests and the lack of proper screening tests. In this study, we developed a deep learning–based screening tool using PGHD obtained from an mHealth app. The experimental results confirm that PGHD from an mHealth app can be a complementary tool for screening for influenza in individual patients. Since our digital approach can screen patients without physical contact, this approach could be quite beneficial in screening new contagious diseases.

## Appendix

Multimedia Appendix 1 App-based surveillance calculated from user input data.

## Abbreviations

AUC: area under the curve
AUROC: area under the receiver operating characteristic
GRU: gated recurrent unit
ILI: influenza-like illness
KCDC: Korea Center for Disease Control
MERS: Middle East respiratory syndrome
mHealth: mobile health
NPV: negative predictive value
PGHD: patient-generated health data
PPV: positive predictive value
RIDT: rapid influenza diagnostic test
ROC: receiver operating characteristic
RT-PCR: reverse transcription-polymerase chain reaction
